# Effect of DR1558, a *Deinococcus radiodurans* response regulator, on the production of GABA in the recombinant *Escherichia coli* under low pH conditions

**DOI:** 10.1186/s12934-020-01322-3

**Published:** 2020-03-10

**Authors:** Sung-ho Park, Yu Jung Sohn, Si Jae Park, Jong-il Choi

**Affiliations:** 1 Department of Biotechnology and Bioengineering, Interdisciplinary Program for Bioenergy & Biomaterials, Chonnam National University, 77 Yongbong-ro, Gwangju, 61186 Republic of Korea; 2 Division of Chemical Engineering and Materials Science, Ewha Womans University, 52 Ewhayeodae-gil, Seodaemun-gu, Seoul, 03760 Republic of Korea

**Keywords:** Gamma aminobutyrate, DR1558, Acid resistance, *Deinococcus radiodurans*, *Escherichia coli*

## Abstract

**Background:**

Gamma aminobutyric acid (GABA) is an important platform chemical, which has been used as a food additive and drug. Additionally, GABA is a precursor of 2-pyrrolidone, which is used in nylon synthesis. GABA is usually synthesized from glutamate in a reaction catalyzed by glutamate decarboxylase (GAD). Currently, there are several reports on GABA production from monosodium glutamate (MSG) or glucose using engineered microbes. However, the optimal pH for GAD activity is 4, which is the limiting factor for the efficient microbial fermentative production of GABA as fermentations are performed at pH 7. Recently, DR1558, a response regulator in the two-component signal transduction system was identified in *Deinococcus radiodurans.* DR1558 is reported to confer cellular robustness to cells by binding the promoter regions of genes via DNA-binding domains or by binding to the effector molecules, which enable the microorganisms to survive in various environmental stress conditions, such as oxidative stress, high osmotic shock, and low pH.

**Results:**

In this study, the effect of DR1558 in enhancing GABA production was examined using two different strategies: whole-cell bioconversion of GABA from MSG and direct fermentative production of GABA from glucose under acidic culture conditions. In the whole-cell bioconversion, GABA produced by *E. coli* expressing GadBC and DR1558 (6.52 g/L GABA from 13 g/L MSG·H_2_O) in shake flask culture at pH 4.5 was 2.2-fold higher than that by *E. coli* expressing only GadBC (2.97 g/L of GABA from 13 g/L MSG·H_2_O). In direct fermentative production of GABA from glucose, *E. coli ∆gabT* expressing isocitrate dehydrogenase (IcdA), glutamate dehydrogenase (GdhA), GadBC, and DR1558 produced 1.7-fold higher GABA (2.8 g/L of GABA from 30 g/L glucose) than *E. coli ∆gabT* expressing IcdA, GdhA, and GadBC (1.6 g/L of GABA from 30 g/L glucose) in shake flask culture at an initial pH 7.0. The transcriptional analysis of *E. coli* revealed that DR1558 conferred acid resistance to *E. coli* during GABA production. The fed-batch fermentation of *E. coli* expressing IcdA, GdhA, GadBC, and DR1558 performed at pH 5.0 resulted in the final GABA titer of 6.16 g/L by consuming 116.82 g/L of glucose in 38 h.

**Conclusion:**

This is the first report to demonstrate GABA production by acidic fermentation and to provide an engineering strategy for conferring acid resistance to the recombinant *E. coli* for GABA production.

## Background

Conventional petroleum-based methods for the production of valuable chemicals are associated with several challenges, such as environmental concerns and limited availability of fossil fuel. Hence, various bio-based production methods using renewable feedstocks have recently been developed [1, 2]. To address the growing demand for the production of value-added products, such as platform chemicals and biopolymers from renewable resources, several engineered microorganisms have been developed [3–9].

Gamma aminobutyric acid (GABA), a non-protein amino acid, is widely expressed in prokaryotic and eukaryotic organisms [10]. GABA is the primary inhibitory neurotransmitter in the nervous system of mammals. Generally, GABA is used in pharmaceuticals or food additives for its physiological functions [11–14]. Additionally, GABA has applications in the bio-based chemical and polymer industries, where GABA is used as the precursor of 2-pyrrolidone, which can be used as a solvent or as a monomer for the synthesis of nylon 4 [15, 16].

Several studies have reported the microbial production of GABA through proton-consuming decarboxylation of l-glutamate using two biological processes: whole-cell bioconversion or direct fermentation. GABA is mainly produced through whole-cell bioconversion of MSG or l-glutamate to GABA by heterologous expression of glutamate decarboxylase (GAD) in the host strain [15, 17–24]. Additionally, several metabolically engineered microorganisms, such as *Corynebacterium glutamicum* and *Escherichia coli* have been developed for the direct fermentative production of GABA from renewable resources, such as glucose and xylose [25–31]. However, there are still several limitations associated with the efficient microbial fermentative production of GABA. For example, the optimal pH for GAD activity is around 4.5, though generally fermentation process of various industrial microorganisms such as *E. coli*, *C. glutamicum,* and *Lactobacillus* is performed around pH 7. These limitations could be addressed by developing GAD able to efficiently convert glutamate into GABA at pH 7 or by developing host strains that supports the enhanced cell robustness under acidic stress for facilitation of the GAD activity. In this regards, the recombinant *C. glutamicum* strain expressing GAD that is active at a wide pH range was reported to produce 38.6 g/L GABA in 72 h fed-batch fermentation from glucose [32]. However, other strategy such as increasing cell tolerance to acidic stress for acidic fermentative production of GABA is but not yet developed.

*Deinococcus radiodurans* is reported to be resistant to various abiotic stress factors, such as γ-radiation, oxidizing agents, and desiccation [33]. Recently, DR1558, a response regulator in the two-component signal transduction system, was identified in *D. radiodurans*. The expression of DR1558, which exhibits a *recA*-like expression pattern, was reported to be enhanced (approximately 5.64-fold) in response to 15 kGy of gamma radiation [34]. DR1558 is reported to induce alterations in the cell by directly binding to the promoter regions of genes via DNA-binding domains or by binding to the effector molecules in response to environmental stress. One study has employed DR1558 for increasing the tolerance of *E. coli* against low pH, heat, and salt stresses [35]. Additionally, DR1558 has been used to enhance cell robustness and production of succinate, 2,3-butanediol (2,3-BDO) and poly(3-hydroxybutyrate) [P(3HB)] in the recombinant *E. coli* strains [36–38].

In this study, the effect of *D. radiodurans* DR1558 on GABA production was examined in the recombinant *E. coli* strains under low pH culture conditions using two different strategies: whole-cell bioconversion of MSG to GABA and direct fermentative production of GABA from glucose. The quantitative real-time polymerase chain reaction (qRT-PCR) analysis was performed to evaluate the expression level of genes related to central metabolism and acid resistance (AR) under acidic challenge in the *dr1558*-overexpressing *E. coli* strains. Furthermore, fed-batch fermentation was carried out for direct fermentative production of GABA from glucose as a proof-of-concept and demonstrated that DR1558 can be robustly used in the fermentation scale.

## Results and discussion

### Examination of DR1558 expression for the acid-resistant conversion of MSG into GABA at low pH

**Fig. 1:**
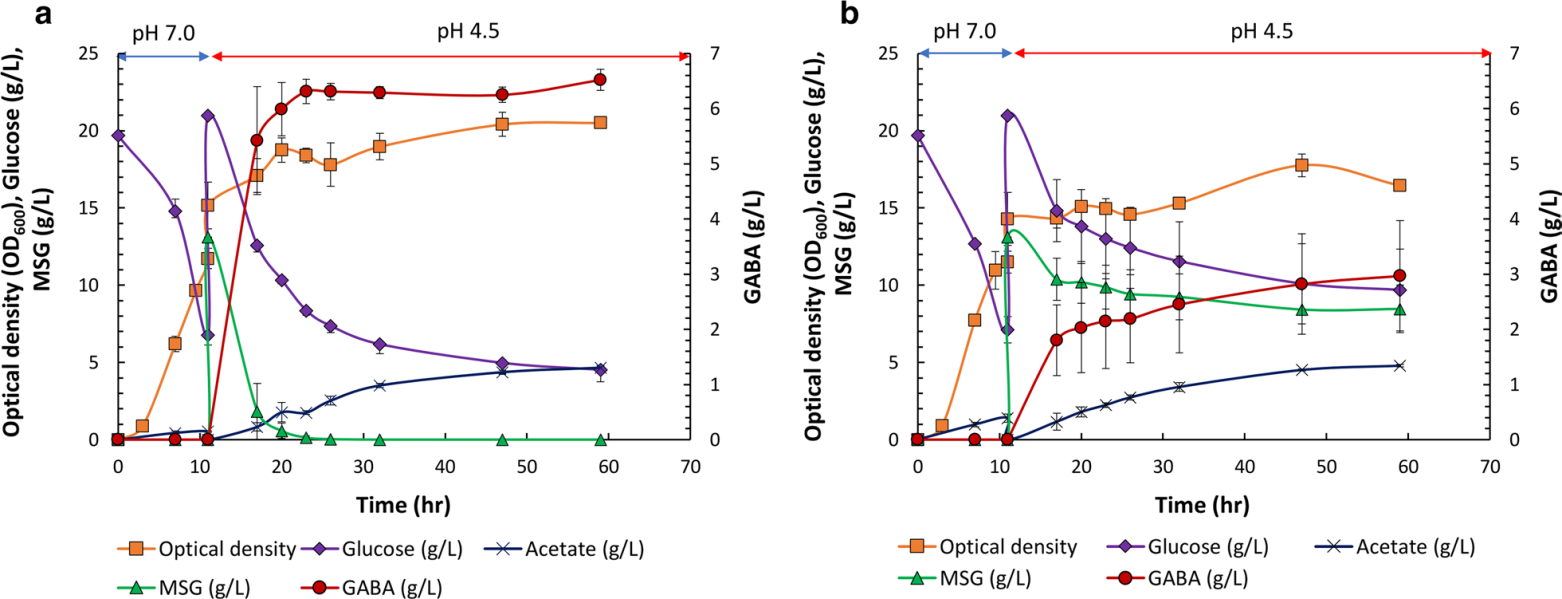
Time profile of growth and production of gamma aminobutyric acid (GABA), monosodium glutamate (MSG), and metabolites in the whole-cell reaction by **a***Escherichia coli* WGB100 strain expressing GadBC and DR1558 and **b***E. coli* BL21(DE3) expressing only GadBC. The strains were cultivated in 50 mL MR medium supplemented with 5 g/L yeast extract, 20 g/L glucose, and 13 g/L MSG·H_2_O in a baffled flask at 30 °C. The data are represented as mean ± standard deviation from three independent experiments

The optimal pH for GAD, which catalyzes the conversion of glutamate into GABA, is around 4.5. This pH is lower than the neutral pH generally used for cell cultivation [39, 40]. Thus, we hypothesized that enhancing *E. coli* resistance to low pH may have a positive effect on GABA production as GABA can be produced at the pH optimum for the decarboxylation activity of GAD. To investigate the effect of DR1558 on GABA production, the recombinant *E. coli* WGB100 strain expressing GadBC and DR1558 and *E. coli* BL21(DE3) strain expressing only GadBC were cultured in the MR medium (pH 7.0) supplemented with 20 g/L glucose and 5 g/L yeast extract. When the optical density at 600 nm (OD_600_) of the culture reached 1.0, isopropyl-β-thiogalactoside (IPTG; final concentration, 0.5 mM) was added to the culture medium to induce protein expression. The cells were cultured for 8 h and transferred to the MR medium (pH 4.5) supplemented with 20 g/L glucose, 5 g/L yeast extract, and 13 g/L MSG·H_2_O. The *E. coli* BL21(DE3) strain expressing only GadBC exhibited a low conversion yield (41%; conversion of 13 g/L MSG·H_2_O into 2.97 g/L GABA) (Fig. 1b). However, the *E. coli* WGB100 strain expressing GadBC and DR1558 exhibited a high GABA yield (87.3%; conversion of 13 g/L MSG·H_2_O into 6.31 g/L GABA) within 24 h. Incubating the *E. coli* WGB100 strain till 60 h resulted in the production of 6.52 g/L GABA corresponding to a 90% yield (Fig. 1a). In previous report, the productivity of 0.12 g GABA/L/h was obtained by recombinant *E. coli* equipped with the C-terminus synthetic complex of GadB and GadC (pH3BN) [41]. Additional expression of DR1558 along with the C-terminus synthetic complex of GadB and GadC in the engineered *E. coli* WGB100 in this study allowed the increased productivity of 0.26 g GABA/L/h [41].

### Transcriptional analysis of genes involved in central metabolism and glutamate acid-dependent acid resistance (GDAR) system in *E. coli*

**Fig. 2:**
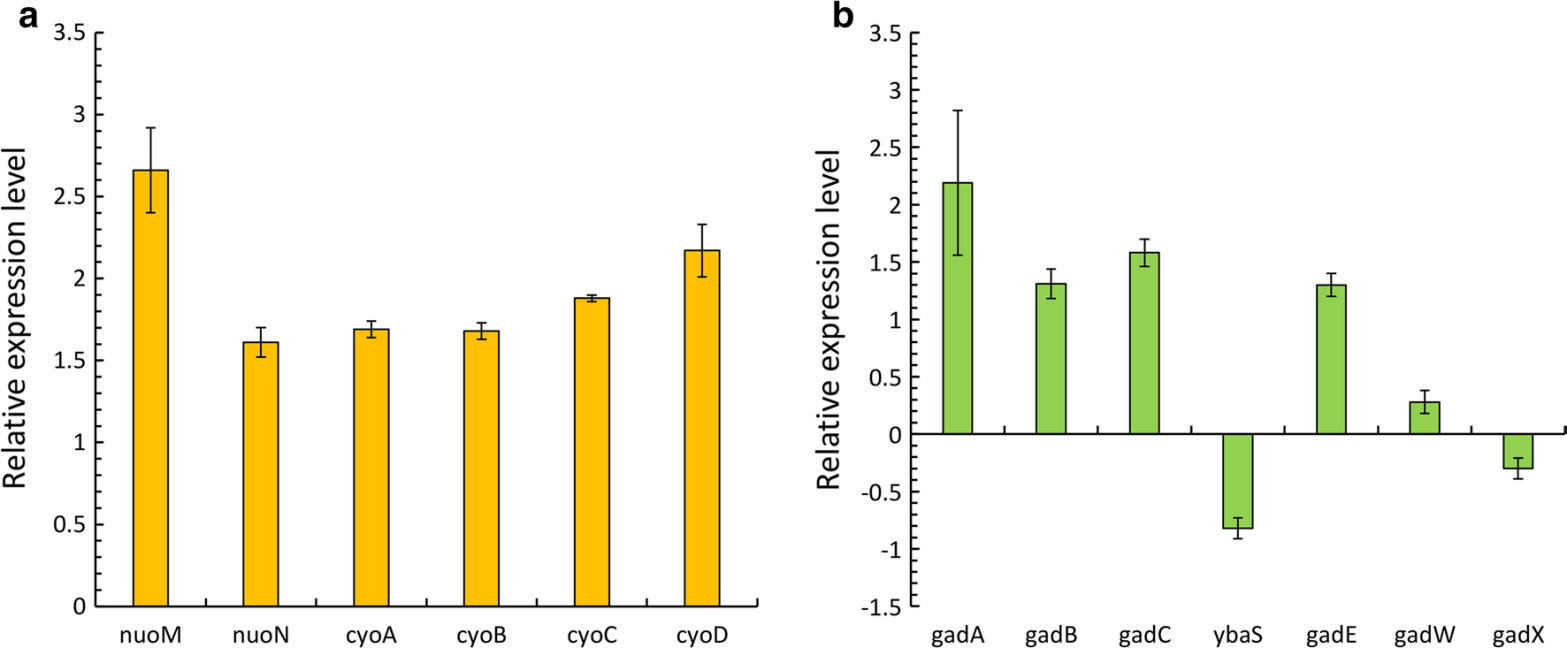
Relative expression of genes related to acid resistance under mild acid stress conditions in *dr1558*-overexpressing *Escherichia coli*. **a** Glutamate-dependent acid resistance (GDAR) system; **b** components of the respiratory chain complexes. Each expression level was converted to the log base 2 of the value. For all genes represented in this figure, the *P*-value was < 0.05 except for *ybaS*, *gadW*, and *gadX*. Histogram shows the mean of three biological replicates and error bars represent standard deviation

The qRT-PCR analysis was performed to analyze the expression patterns of 37 genes associated with the central carbon metabolism, AR system, and respiratory chain complexes in the *E. coli* WGB100 and control strains as summarized in Fig. 2 and Additional file 1: Table S2.

When the pH was decreased, the expression levels of genes involved in the central metabolic pathways, such as glycolysis and tricarboxylic acid (TCA) cycle in the *E. coli* WGB100 strain were upregulated when compared to those in the control strain (Additional file 1: Table S2). The expression levels of *ptsG* and *pykA*, which are associated with the glycolytic pathway, in the *E. coli* WGB100 strain were upregulated by 2.1-, and 1.76-fold, respectively, when compared to the control strain. This indicated enhanced glucose uptake by the *E. coli* WGB100 strain. As shown in Fig. 1, although the production of acetate in the recombinant *E. coli* WGB100 strain expressing both GadBC and DR1558 was similar to that of the control strain, the glucose consumption increased by 1.51-fold. This suggested that the carbon flux towards acetate metabolism in the *E. coli* WGB100 strain decreased based on the production of acetate relative to the consumption of carbon source. The expression of the *poxB* gene, which is involved in acetate metabolism, was downregulated by 3.67-fold in the *E. coli* WGB100 strain when compared to its expression in the control strain (Additional file 1: Table S2). The downregulation of acetate metabolism was also observed in our previous study in the *dr1558*-overexpressing *E. coli* for the production of 2,3-BDO and P(3HB) [37, 38].

Under low pH conditions, *E. coli* can maintain the pH homeostasis of the intracellular environment via carboxylation of amino acid by the proton-consuming AR system [42, 43]. *E. coli* contains four different amino acid-dependent AR systems: GDAR, arginine-dependent acid resistance system (ADAR), lysine-dependent acid resistance system (LDAR), and ornithine-dependent acid resistance system (ODAR). Among these four systems, the GDAR system comprising GadA, GadB, and GadC is associated with low pH tolerance in the *dr1558*-overexpressing *E. coli* [35, 44–48]. Hence, the expression pattern of genes involved in the GDAR system was examined. The expression levels of *gadA*, *gadB*, and *gadC* genes in the *E. coli* WGB100 strain were upregulated by 2.19-, 1.13-, and 1.58-fold when compared to those in the control strain, respectively (Fig. 2b). Moreover, the mRNA levels of the *rpoS* and *gadE* genes, which indirectly affect the GDAR system, were also examined (Additional file 1: Table S2 and Fig. 2b). The proteins encoded by these genes, especially GadE, are involved in transcriptional regulation. GadE is the main regulator of the GDAR system and other acid fitness islands (AFIs), while RpoS is a sigma factor that simulates GadE expression [49, 50]. The transcriptional analysis revealed upregulated expression levels of *rpoS* and *gadE* genes, which subsequently increased the expression level of GDAR system.

Along with the GDAR system, various respiratory chain complexes, such as cytochrome *b*_*0*_ oxidase (Cyo), succinate dehydrogenase (SDH), and NADH dehydrogenase I (Nuo) that pump protons out of the cell are activated in *E. coli* as a metabolic response to acidic stress [43]. Cyo exports cytoplasmic protons by converting molecular oxygen to H_2_O to generate the proton motive force (PMF) during aerobic growth under acidic conditions. Additionally, Nuo catalyzes the oxidation of NADH to NAD, which contributes to PMF by directly pumping out the protons [47, 51]. Hence, we also examined the expression level of genes associated with respiratory chain complexes. The expression levels of *nuoMN* and *cyoABCD* in the *dr1558*-overexpressing strain were 1.5-fold higher than those in the control strain (Fig. 2a). Thus, overexpression of DR1558 resulted in enhanced expression of GDAR system as well as the respiratory chain complexes, which improved cell viability of *E. coli* WGB100 by conferring acid resistance during the conversion of MSG to GABA at low pH culture conditions.

### Reconstruction of GABA production pathway for the direct production of GABA from glucose

**Fig. 3:**
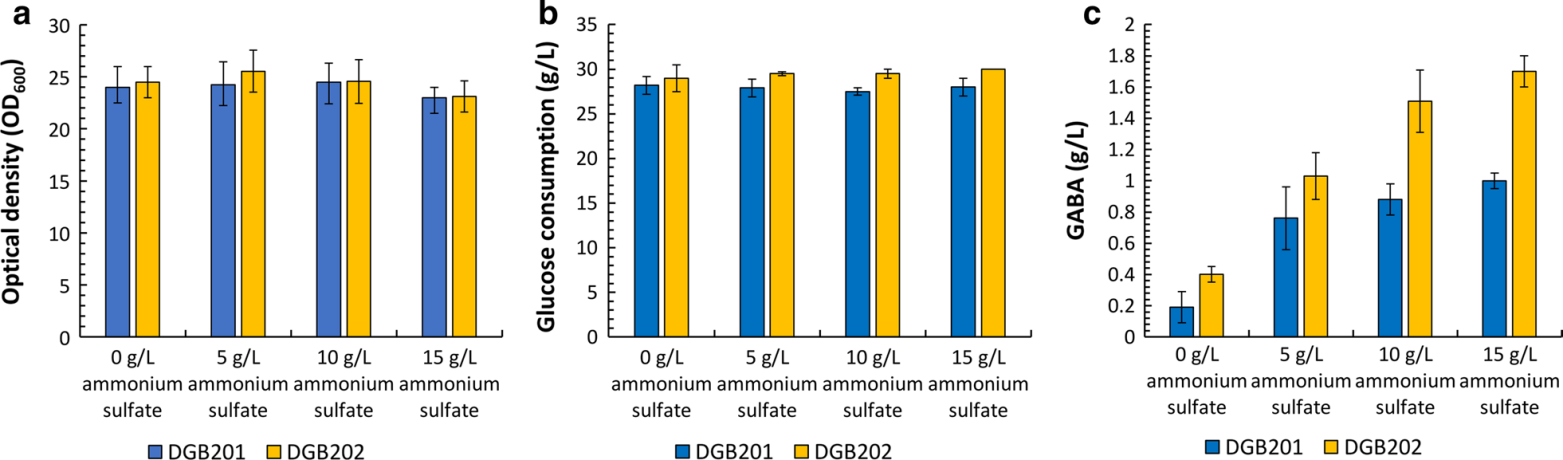
Effect of ammonium sulfate supplementation on cell growth, glucose consumption, and gamma aminobutyric acid (GABA) production. The *Escherichia coli* DGB201 and DGB202 strains were cultured in MR medium supplemented with 30 g/L glucose, 5 g/L yeast extract, and (0, 5, 10, or 15 g/L) ammonium sulfate. **a** Cell growth; **b** glucose consumption; **c** GABA production. Histogram shows the mean of three biological replicates and error bars represent standard deviation

The *E. coli* WGB100 strain can convert MSG into GABA while consuming glucose at pH 4.5. Hence, we hypothesized that GABA could be produced directly from glucose at low pH culture conditions via production of glutamate, a direct precursor of GABA (Fig. 1). Therefore, we constructed the metabolic pathway for direct synthesis of GABA from glucose. In a previous study, enhanced expression of *icdA* encoding isocitrate dehydrogenase and *gdhA* encoding glutamate dehydrogenase was reported to enhance the production of l-glutamate in the engineered *E. coli* [52]. Thus, the two *E. coli* BL21(DE3)-derived mutant strains, *E. coli* DGB201 (BL21(DE3) *∆gabT* expressing GdhA and, GadBC) and *E. coli* DGB202 (BL21(DE3) *∆gabT* expressing IcdA, GdhA and, GadBC), were investigated for the production of GABA from glucose (Table 1). The initial pH in the culture medium was set to 7.0 and the pH was not adjusted during the cultivation. After the formation of various organic acids, such as acetate and lactate, resulted in decreasing the pH in the culture medium, GABA was produced from glucose by the *E. coli* DGB201 and DGB202 strains (Additional file 1: Figure S1). However, the *E. coli* DGB201 and DGB202 strains exhibited a low production of GABA (up to 0.19 and 0.4 g/L, respectively) from 30 g/L of glucose when they were cultured in the medium without additional inorganic nitrogen source at an initial pH of 7.0 (Fig. 3c). To produce GABA from glucose efficiently, the production of glutamate should be properly supported by the recombinant *E. coli* host strain. Previous studies have reported that the supplementation of inorganic nitrogen sources, such as ammonium sulfate, urea, and ammonium chloride in the culture medium improved the production of l-amino acid by the microbial host strain [53]. Hence, ammonium sulfate was added to the culture medium containing 30 g/L glucose to examine its effect on the production of GABA. The cell growth and glucose consumption in both the *E. coli* DGB201 and DGB202 strains were not markedly affected with supplementation of ammonium sulfate at concentrations of up to 15 g/L (Fig. 3a, b). Interestingly, glutamate was not detected in any of the culture media and GABA production was proportional to the concentration of ammonium sulfate in the culture medium. The supplementation of 15 g/L ammonium sulfate resulted in the highest GABA concentrations of 1.7 and 1 g/L in the *E. coli* DGB202 and *E. coli* DGB201 strain, respectively (Fig. 3c).

**Table 1:** Plasmid, strain used in this study

Plasmids, strains	Characteristics	Origin/Ab^R^	Source
Plasmid			
pACYCDuet-1	P_T7_::MSC	p15A/Cm^R^	Novagen
pH3BN	P_tac_::*gadB*-SH3D, *gadC*-SH3L	ColE1/Amp^R^	[41]
pGB100	P_T7_::*dr1558*	p15A/Cm^R^	This study
pGB101	P_T7_::*gdhA*-T_T7_	p15A/Cm^R^	This study
pGB102	P_T7_::*icdA*, *gdhA*-T_T7_	p15A/Cm^R^	This study
pGB103	P_T7_::*dr1558*-P_T7_::*icdA*, *gdhA*-T_T7_	p15A/Cm^R^	This study
pREDET	P_BAD_::*gam*, *beta*, *alpha*, *recA*	pSC101/Amp^R^	Gene Bridges
pMloxC	lox66-Cm-lox71 cassette	Co1E1/Amp^R^, Cm^R^	[54]
pJW168	Cre-recombinase	pSC101/Amp^R^	[55]
Strain			
BL21(DE3)	*fhuA2 [lon] ompT gal (λ DE3)[dcm] ∆hsdS*		NEB
GB200	BL21(DE3) *ΔgabT*		This study
GB300	BL21(DE3) *ΔgabT, ΔsucA*		This study
WGB100	BL21(DE3)/pGB100, pH3BN		This study
WGB200	GB200/pGB100, pH3BN		This study
DGB101	BL21(DE3)/pGB101, pH3BN		This study
DGB102	BL21(DE3)/pGB102, pH3BN		This study
DGB201	GB200/pGB101, pH3BN		This study
DGB202	GB200/pGB102, pH3BN		This study
DGB203	GB200/pGB103, pH3BN		This study
DGB301	GB300/pGB101, pH3BN		This study
DGB302	GB300/pGB102, pH3BN		This study
DGB303	GB300/pGB103, pH3BN		This study

### Effect of DR1558 on direct production of GABA from recombinant *E. coli*

The effect of DR1558 on the production of GABA was examined in the *E. coli* DGB203 (BL21(DE3) *∆gabT* expressing IcdA, GdhA, GadBC, and DR1558) strain. The *E. coli* DGB202 (BL21(DE3) *∆gabT* expressing IcdA, GdhA, and GadBC) strain was used as a control. Both *E. coli* strains were cultured in the MR medium supplemented with 30 g/L glucose, 5 g/L yeast extract, and 15 g/L ammonium sulfate at an initial pH of 7.0 for 24 h. When the cultures reached an OD_600_ of 0.5, 0.5 mM IPTG was added to induce the GABA production pathway.

**Fig. 4:**
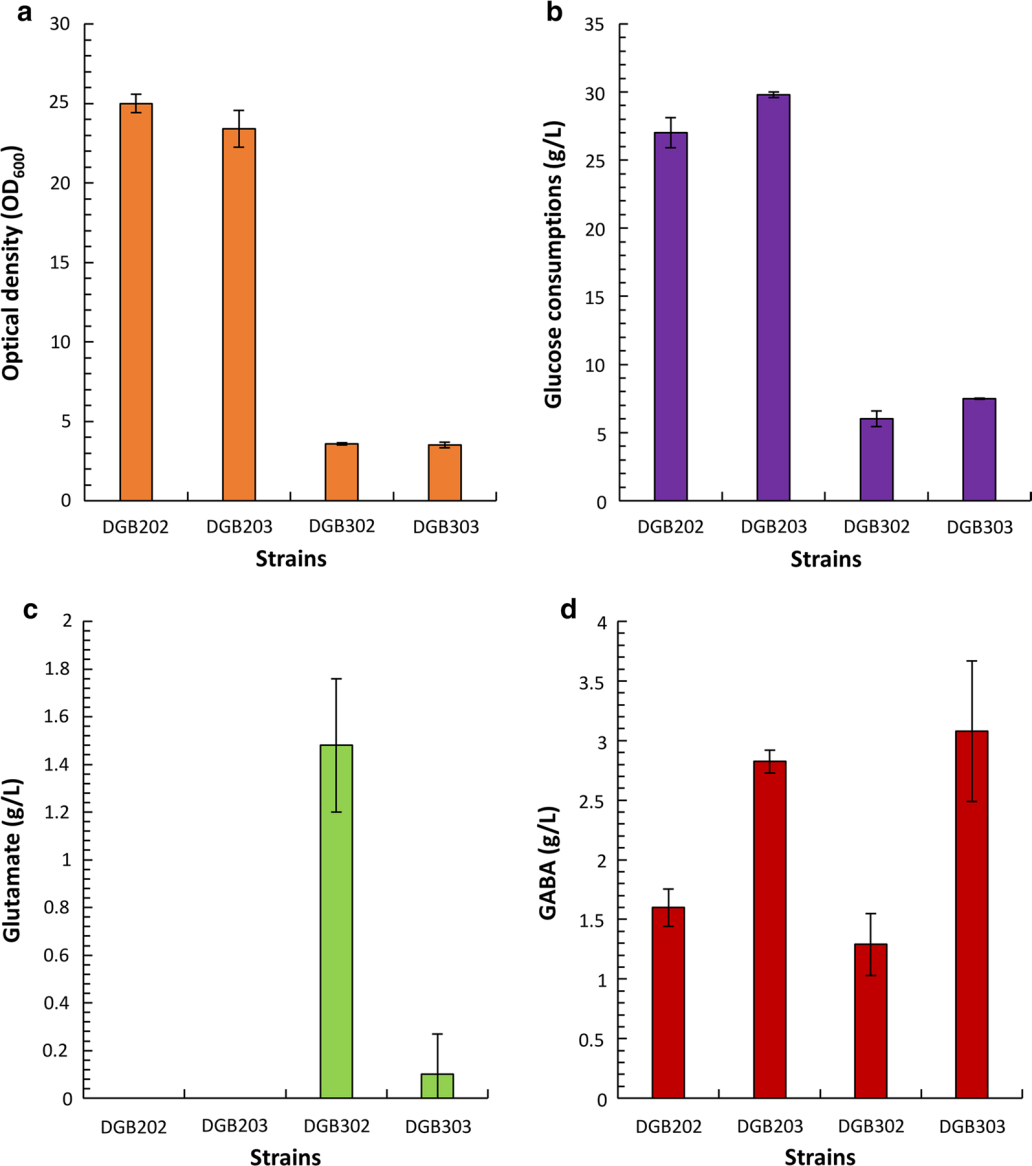
Effect of DR1558 on cell growth, glucose consumption, glutamate production, and gamma aminobutyric acid (GABA) production in the recombinant *Escherichia coli*. The *E. coli* DGB202 and DGB203 strains were cultured in MR medium supplemented with 5 g/L yeast extract, 30 g/L glucose, and 15 g/L ammonium sulfate for 24 h. The *E. coli* DGB302 and DGB303 strains were cultured in MR medium supplemented with 5 g/L yeast extract, 20 g/L glucose, and 10 g/L ammonium sulfate for 48 h. **a** Cell growth; **b** glucose consumption; **c** glutamate production; **d** GABA production. Histogram shows the mean of three biological replicates and error bars represent standard deviation

The *E. coli* DGB203 strain exhibited a higher glucose consumption than the *E. coli* DGB202 strain (Fig. 4b). The final cell density of the *E. coli* DGB202 strain was higher than that of the *E. coli* DGB203 strain (Fig. 4a). Although glutamate was not detected in both *E. coli* DGB202 and DGB203 strains during cultivation, the titer and yield of GABA production in the *E. coli* DGB203 strain increased from 1.6 to 2.8 g/L and 0.059 to 0.093 (g_GABA_/g_Glucose_), respectively, when compared to those in the *E. coli* DGB202 strain (Fig. 4c, d).

As the yields of GABA obtained in both *E. coli* DGB202 and DGB203 strains were low, *sucA* encoding 2-oxoglutarate decarboxylase, which competes with *gdhA* encoding glutamate dehydrogenase in the GABA pathway, was deleted in the *E. coli* GB200 (BL21(DE3) *∆gabT*) strain. The GABA titer in the *E. coli* DGB303 (BL21(DE3) *∆gabT ∆sucA* expressing IcdA, GdhA, GadBC, and DR1558) strain slightly increased from 2.82 to 3.08 g/L when compared to that in the *E. coli* DGB203 strain. Additionally, the yield of GABA in the *E. coli* DGB303 strain significantly increased from 0.093 to 0.41 (g_GABA_/g_Glucose_), corresponding to a 4.41-fold increase, when compared to that in the *E. coli* DGB203 strain (Fig. 4d). However, the productivity of GABA production in the *E. coli* DGB303 strain decreased from 0.12 to 0.064 (g/L/h) when compared to that in the *E. coli* DGB203 strain. As *sucA* is an essential gene for *E. coli* cell growth, the final cell density of *E. coli* DGB303 was lower than that of *E. coli* DGB203. Therefore, *E. coli* DGB203 strain was selected for further experiments.

Interestingly, the *E. coli* DGB302 (BL21(DE3) *∆gabT ∆sucA* expressing IcdA, GdhA, and GadBC genes) strain failed to convert glutamate into GABA and secreted 1.13 g/L glutamate into the culture medium, whereas the *E. coli* DGB303 strain did not secrete glutamate into the culture medium (Fig. 4c, d).

### Production of GABA from glucose in fed-batch culture

**Fig. 5:**
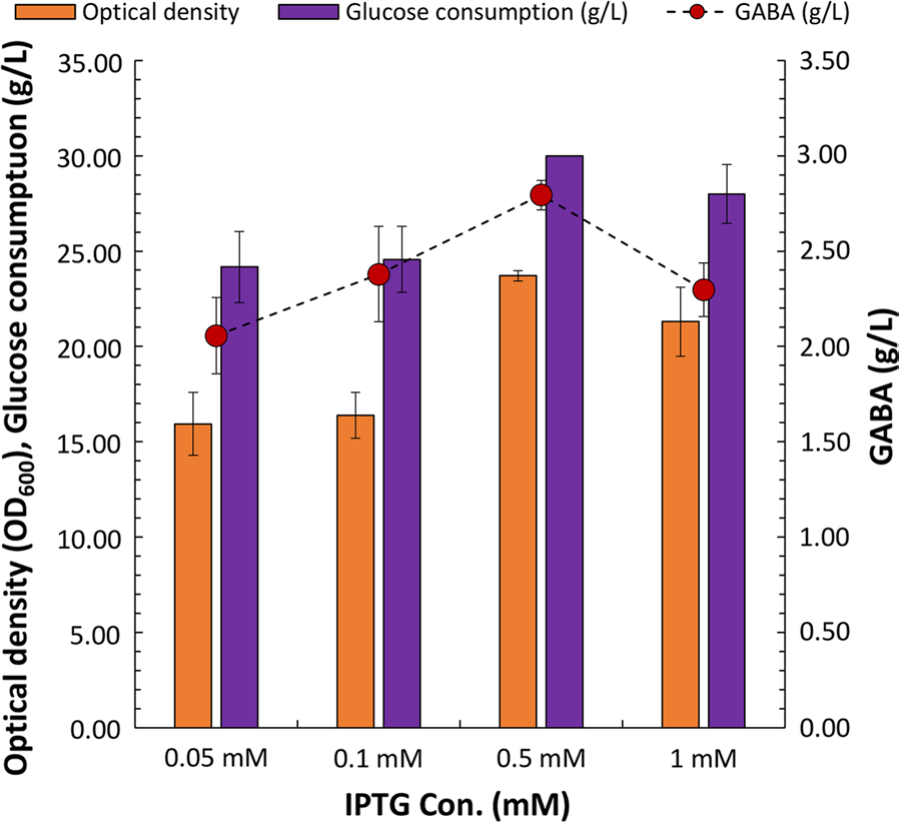
Effect of isopropyl-β-thiogalactoside (IPTG) on cell growth, glucose consumption, and gamma aminobutyric acid (GABA) production. The *Escherichia coli* DGB203 strain was cultured in MR medium supplemented with 5 g/L yeast extract, 30 g/L glucose, and 15 g/L ammonium sulfate for 24 h. Histogram shows the mean of three biological replicates and error bars represent standard deviation

To optimize the concentration of IPTG in the culture medium, different concentrations of IPTG (0.05, 0.1, 0.5, or 1 mM) were supplemented in the MR medium with a glucose/ammonium sulfate ratio of 2. The cell growth in the medium supplemented with 0.05 and 0.1 mM IPTG significantly decreased (from 23 to 16 of OD_600_) when compared to that in the medium supplemented with 0.5 mM IPTG. However, in all concentrations of IPTG except for 1 mM, the titer of GABA was increased by the addition of IPTG. The highest titer of GABA (2.79 g/L) was obtained in the medium supplemented with 0.5 mM IPTG (Fig. 5). Based on these results, the fed-batch culture of the *E. coli* DGB203 strain was performed in the MR medium with a glucose/ammonium sulfate ratio of 2 and 0.5 mM IPTG.

**Fig. 6:**
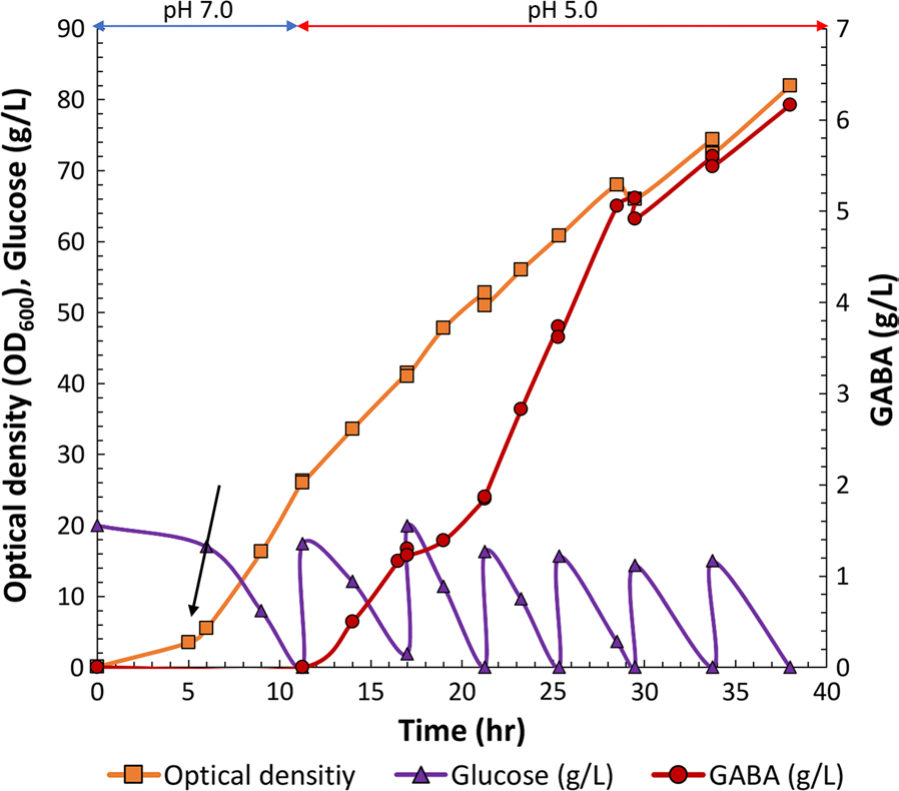
The fermentation profile during the synthesis of gamma aminobutyric acid (GABA) from glucose by the *Escherichia coli* DGB203 strain in fed-batch culture. The black arrow represents induction timing for gene expression

The cells were cultured till OD_600_ of 5 at pH 7.0 and IPTG was added to the culture medium at a final concentration of 0.5 mM to induce protein expression. The cells were further cultured for 5 h at pH 7.0 and then the pH was lowered to 5.0. GABA was initially not detected in the culture medium at pH 7.0 after induction. However, decreasing the pH to 5.0 and the addition of glucose enhanced the GABA production. The cell density reached an OD_600_ of 82 in 38 h. The titer of GABA reached 6.16 g/L after 38 h and the glucose consumption was 116.82 g/L. During fed-batch fermentation, glutamate and fermentative by-products, such as acetate and lactate were not detected in the culture medium (Fig. 6). These results indicated that DR1558 was beneficial for the carbon flux toward GABA production under acidic environmental conditions at the fermentation scale.

Currently, there are several reports on direct fermentative production of GABA from glucose using engineered *E. coli*. The recombinant *E. coli* containing the synthetic scaffolds between *icdA*, *gltB*, and *gadB* produced 1.3 g/L of GABA from 10 g/L glucose in 48 h [25]. Additionally, GABA production from glucose through GABA shunt in the recombinant *E. coli* was 1.08 g/L from 10 g/L glucose in 48 h [30]. Recently, synthetic metabolic toggle switch, which controls the expression of multiple metabolic states, was introduced in *E. coli* to produce GABA from glucose, which resulted in the production of 4.8 g/L GABA from 20 g/L glucose [56]. However, these GABA fermentation processes were performed in the shake flask scale. In this study, we demonstrated that introducing DR1558 in the GABA-producing *E. coli* provides robustness to cells against acid stress for fermentative scale production of GABA from glucose under acid culture conditions. Thus, this study provides an engineering strategy for GABA production from glucose in industrial processes under low pH condition.

## Conclusions

**Fig. 7:**
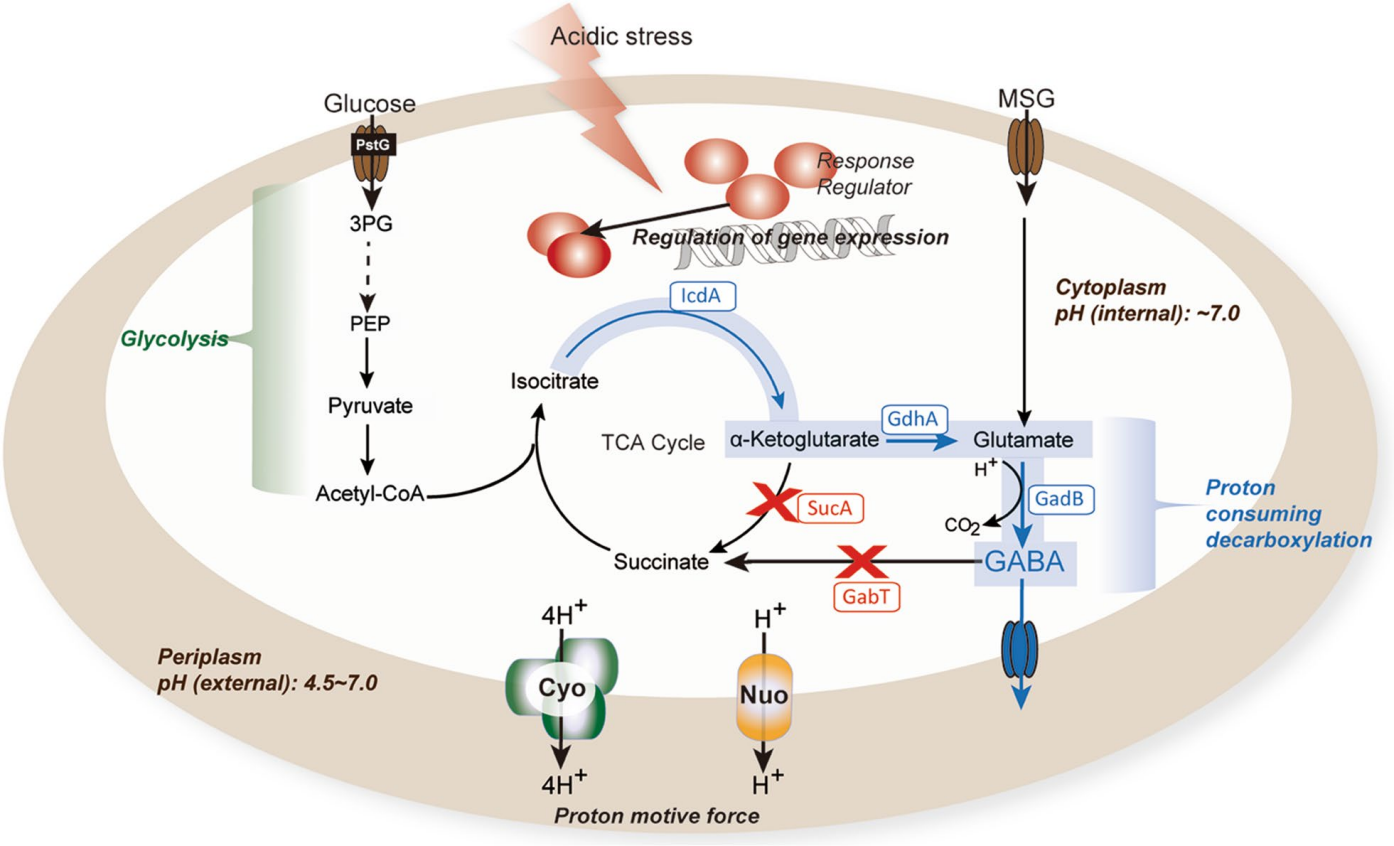
Metabolic engineering of *E. coli* for the production of gamma aminobutyric acid (GABA) using a response regulator. The blue line represents gene overexpression. The red X's represents knockout of genes

In this study, we reported that DR1558 can enhance the production of GABA at low pH, optimal for GAD which had been a limiting factor for fermentative GABA production in the recombinant *E. coli*. We developed two different GABA production strategies: whole-cell bioconversion of GABA from MSG and direct fermentative production of GABA from glucose. Under low pH culture conditions, the *dr1558*-overexpressing *E. coli* exhibited enhanced cell growth rate and GABA production. Furthermore, the transcriptional analysis revealed that DR1558 enabled the growth of *E. coli* at pH 4.5 by increasing the expression levels of GDAR system and proton-pumping respiratory chain complexes. As the bioconversion of GABA from glutamate was observed with optimal cell viability under acidic conditions by the introduction of DR1558, fermentative production of GABA from glucose was also examined. The fermentative process enhanced the production of GABA with the expression of *dr1558* in *E. coli* (Fig. 7). To the best of our knowledge, this is the first study to report the acidic fermentation of GABA. This study provides an engineering strategy for conferring AR to *E. coli*, which can be used to produce GABA via the GAD system that functions at low pH.

## Methods

### Bacterial strains and plasmids

All bacterial strains, plasmids, and primers used in this study are listed in Table 1 and Additional file 1: Table S1. In this study, *E. coli* XL1-Blue (Stratagene Cloning Systems, La Jolla, CA, USA) was used for standard gene cloning. For the synthesis of GABA, *E. coli* BL21 (DE3) (New England Biolabs, Ipswich, MA, USA) and its mutants were used as host strains.

The *E. coli* GB200 (BL21(DE3) *ΔgabT*) and GB300 (BL21(DE3) *ΔgabT ΔsucA*) strains were constructed by deleting the corresponding genes in the chromosome of *E. coli* BL21(DE3) using the single-step gene knockout method as previously described [57].

The construction of pH3BN plasmid expressing *N. crassa gadB* and *E. coli gadC*, which convert glutamate into GABA, was previously described [41]. The pGB100 plasmid was constructed by inserting the *dr1558* gene, which was amplified by PCR using the dr1558-F and dr1558-R primers, from the genomic DNA of *D. radiodurans* into the pACYCDuet-1 (Novagen, Madison, WI, USA) plasmid at the NcoI and BamHI restriction sites.

The pGB101, pGB102, and pGB103 plasmids were constructed based on the pACYCDuet-1 plasmid to synthesize endogenous glutamate, a precursor of GABA, from glucose. The pGB101 plasmid was constructed by inserting the *E. coli gdhA* gene into the pACYCDuet-1 plasmid at the *Eco*RV and *Xho*I restriction sites. The pGB102 plasmid was constructed by inserting the *E. coli icdA* gene into the pGB101 plasmid at the NdeI and BglII restriction sites. Finally, the *dr1558* gene was inserted into the pGB102 plasmid at the NcoI and BamHI restriction sites to generate the pGB103 plasmid.

### Culture condition for whole-cell bioconversion of glutamate to GABA

The *E. coli* WGB100 strain was used as a host strain for whole-cell bioconversion of glutamate to GABA. The seed cultures were cultivated at 30 °C in a 10-mL tube containing 3 mL Luria–Bertani (LB) medium (10 g/L tryptone, 5 g/L yeast extract, and 5 g/L NaCl) for 12 h at 250 rpm. For shake flask cultures, 3% (v/v) seed cultures were added to 50 mL MR medium supplemented with 20 g/L glucose and 5 g/L yeast extract. The composition of the MR medium (pH 7.0) was as follows: 6.67 g/L KH_2_PO_4_, 4 g/L (NH_4_)_2_HPO_4_, 0.8 g/L MgSO_4_·7H_2_O, 0.8 g/L citric acid, and 5 mL/L of trace metal solution. The composition of trace metal solution was as follows: 10 g/L FeSO_4_·7H_2_O, 2 g/L CaCl_2_, 2.2 g/L ZnSO_4_·7H_2_O, 0.5 g/L MnSO_4_·4H_2_O, 1 g/L CuSO_4_·5H_2_O, 0.1 g/L (NH_4_)_6_Mo_7_O_24_·4H_2_O, and 0.02 g/L Na_2_B_4_O_7_·10H_2_O prepared in 0.5 M HCl. MgSO_4_·7H_2_O was sterilized separately. Ampicillin (Ap, 100 μg/mL) and chloramphenicol (Cm, 35 μg/mL) were added to the medium depending on the antibiotic resistance marker of plasmids. When the OD_600_ of the culture reached 1.0, IPTG was added to the culture medium at a final concentration of 0.5 mM to induce protein expression. The cells were cultured for 8 h after IPTG induction. The recombinant *E. coli* strain was harvested by centrifugation at 8000*g* and 4 °C for 10 min. The cell pellet was washed with distilled water to remove the salts. The cells were then transferred to fresh MR (pH 4.5) medium supplemented with 20 g/L glucose, 5 g/L yeast extract, and 13 g/L MSG·H_2_O (Dae Jung Chemicals and Metals Co. Ltd., Siheung, Korea) for the production of GABA.

### Culture condition for direct synthesis of GABA from glucose

For the direct synthesis of GABA from glucose, the recombinant *E. coli* DGB201, DGB202, and DGB203 strains were cultured in MR medium (pH 7.0) supplemented with 30 g/L glucose, 5 g/L yeast extract, and 15 g/L ammonium sulfate at 30 °C for 24 h. The recombinant *E. coli* DGB302 and DGB303 strains were cultured in the MR medium (pH 7.0) supplemented with 20 g/L glucose, 5 g/L yeast extract, and 10 g/L ammonium sulfate at 30 °C for 48 h. Next, 35 μg/mL chloramphenicol and 100 μg/mL ampicillin were added to the culture medium. When the OD_600_ of the culture reached 0.5, IPTG was added to the culture medium at a final concentration of 0.5 mM.

The fed-batch culture was performed at 30 °C in a 5-L bioreactor (BioCNS, Daejeon, Korea) containing 1.8 L of MR medium supplemented with 5 g/L yeast extract, 20 g/L glucose, and 10 g/L ammonium sulfate. The seed culture (200 mL) was prepared in LB medium. When the OD_600_ of the culture reached 5, IPTG was added to the culture medium at a final concentration of 0.5 mM for inducing protein expression. The culture pH was adjusted using 5 M NaOH and 5 M HCl. The level of dissolved oxygen concentration (DOC) was maintained at 20% by automatically increasing the agitation speed to 1000 rpm and supplying pure oxygen. The feeding nutrition solutions were added to the culture medium when glucose levels depleted. The composition of the feeding nutrition was as follows: 500 g/L glucose, 500 g/L ammonium sulfate, 400 g/L yeast extract, 150 g/L MgSO_4_·7H_2_O. For the GABA production, the culture pH was adjusted to 5.0 using 5 M HCl when the initially supplied glucose levels depleted.

### Analytical procedure

The cell growth was analyzed by measuring the optical density (OD_600_) using a UV spectrophotometer (Molecular Devices, San Jose, CA). To determine the GABA and glutamate contents, the cell supernatants were analyzed by high-performance liquid chromatography (HPLC, Agilent Technologies Inc., Santa Clara, CA) equipped with ZORBAX SB-C18 column (250 × 4.6 mm) (Agilent Technologies Inc., Santa Clara, CA) as described previously [58]. The concentrations of organic acids and glucose were analyzed by HPLC equipped with Aminex HPX-76H column (300 × 7.8 mm) (Bio-Rad, Hercules, CA).

### qRT-PCR

To investigate the changes in the gene expression pattern, the cells were cultured for 3 h after the pH was reduced to 4.5. The cells were then harvested by centrifugation at 10,000*g* and 4 °C for 5 min (Fig. 1). Total RNA was extracted using the RNA mini prep kit (Qiagen, Venlo, The Netherlands), following the manufacturer’s instructions. The isolated RNA was reverse transcribed using the first-strand cDNA synthesis kit (TaKaRa Bio Inc., Kusatsu, Japan), following the manufacturer’s instructions. The primers used in the qRT-PCR analysis are described in Additional file 1: Table S1. To analyze the transcriptional level, 20 ng of template cDNA and 10 pmol of each primer were added to the TB green premix Ex Taq (TaKaRa) in a Real-Time PCR system (Illumina Inc., San Diego, CA). The PCR conditions were as follows: 40 cycles of 95 °C for 10 s and at 58 °C for 30 s. The data were log_2_-transformed and normalized using 2^−ΔΔCt^ method for the analysis of the relative changes in gene expression. When using the 2^−ΔΔCt^ method, the transcriptional results are presented as the fold changes in gene expression normalized to the internal control and relative to the untreated control [59]. The internal controls, used in qRT-PCR for the purpose of normalizing the PCRs, were *polA* and *dnaA*, which encode DNA polymerase I and dual-transcriptional regulator, respectively. Transcriptional analysis of *E. coli* WGB100 (pGB100 and pH3BN) as *dr1558* overexpressing strain and *E. coli* (pH3BN) as control strain was carried out to elucidate how DR1558 affects the metabolic pathway of host cell in GABA production. All experiments were performed in triplicates. The statistical significance of the observed differences in gene expression was evaluated by a student’s *t*-test. Genes whose expression differed more than twofold were considered differentially expressed. Only changes with a threshold *P*-value of < 0.05 were seen as statistically significant.

## Supplementary information

**Additional file 1: Table S1.** Primers used in this study. **Table S2.** Relative gene expression of *dr1558* overexpression strain. **Figure S1.** Time profile of GABA and pH in flask cultivation of recombinant *E. coli.* A) *E. coli* DGB201 strain expressing GdhA and GadBC genes.; B) *E. coli* DGB202 strain expressing IcdA, GdhA, and GadBC genes. Strains were cultivated in 50 mL MR medium supplied with 5 g/L Yeast Extract, 30 g/L glucose in a baffled flask at 30 °C for 24 h.

## Data Availability

Please contact corresponding author for data requests.

## Abbreviations

*aceA* (Icl): Isocitrate lyase
*aceB* (MS): Malate synthase
*aceE* (AceE): Pyruvate dehydrogenase E1 component
*ackA* (AckA): Acetate kinase
ACOA: Acetyl-CoA
*acs* (Acs): Acetyl-CoA synthetase
*actP* (ActP): Acetate permease
*crp* (Crp): cAMP receptor protein
*cyoABCD* (CyoABCD): Cytochrome *b*_*0*_ ubiquinol oxidase
*fruR* (FruR): Catabolite repressor
G6P: Glucose 6-phosphate
*gadA* (GadA): Glutamate decarboxylase A
*gadB* (GadB): Glutamate decarboxylase B
*gadC* (GadC): Glutamate/gamma-aminobutyrate antiporter
*gadE* (GadE): DNA-binding transcriptional activator
*gadW* (GadW): DNA-binding transcriptional dual regulator
*gadX* (GadX): DNA-binding transcriptional dual regulator
*icdA* (IcdA): Isocitrate dehydrogenase
*ihfA* (IHF α subunit): Subunit of integration host factor
*mdh* (Mdh): Malate dehydrogenase
*nuoMN* (NuoMN): Subunit of NADH:quinone oxidoreductase I
OAA: Oxaloacetate
*pdhR* (PdhR): Pyruvate dehydrogenase complex repressor
PEP: Phosphoenolpyruvate
*poxB* (PoxB): Pyruvate oxidase
*pta* (Pta): Phosphotransacetylase
*ptsG* (PtsG): Subunit of enzyme IIglc
*pykA*, *pykF* (Pyk): Pyruvate kinase
PYR: Pyruvate
*rpoD* (RpoD): RNA polymerase sigma 70 subunit
*rpoS* (RpoS): RNA polymerase sigma 38 subunit
*sdhC* (Sdh): Succinate dehydrogenase
*sucA* (SucA): 2-oxoglutarate dehydrogenase E1 component
*ybaS* (YbaS): Glutaminase A
*zwf* (Zwf): Glucose 6-phosphate dehydrogenase
α-KG: Alpha ketoglutarate

## References

[1] Kim HT, Baritugo K, Hyun SM, Khang TU, Sohn YJ, Kang KH, Jo SY, Song BK, Park K, Kim IK, Hwang YT, Lee SY, Park SJ, Joo JC (2020). Development of metabolically engineered *Corynebacterium glutamicum* for enhanced production of cadaverine and its use for the synthesis of bio-polyamide 510. ACS Sus Chem Eng.

[2] Oh YH, Eom IY, Joo JC, Yu JH, Song BK, Lee SH, Hong SH, Park SJ (2015). Recent advances in development of biomass pretreatment technologies used in biorefinery for the production of bio-based fuels, chemicals and polymers. Korean J Chem Eng.

[3] Pang B, Valencia LE, Wang J, Wan Y, Lal R, Zargar A, Keasling JD (2019). Technical advances to accelerate modular type I polyketide synthase engineering towards a retro-biosynthetic platform. Biotechnol Bioprocess Eng.

[4] Kim HT, Baritugo K, Oh YH, Hyun SM, Khang TU, Kang KH, Jung SH, Song BK, Park K, Kim IK, Lee MO, Kam Y, Hwang YT, Park SJ, Joo JC (2018). Metabolic engineering of *Corynebacterium glutamicum* for the high-level production of cadaverine that can be used for the synthesis of bio-polyamide 510. ACS Sustain Chem.

[5] Kim HT, Kim JK, Cha HG, Kang MJ, Lee HS, Khang TU, Yun EJ, Lee DH, Song BK, Park SJ, Joo JC, Kim KH (2019). Biological valorization of polyethylene terephthalate monomers for waste upcycling. ACS Sustain Chem.

[6] Rhie MN, Kim HT, Jo SY, Chu LL, Baritugo KA, Baylon MG, Lee J, Na JG, Kim LH, Kim TW, Park C, Hong SH, Joo JC, Park SJ (2019). Recent advances in the metabolic engineering of *Klebsiella pneumoniae*: a potential platform microorganism for biorefineries. Biotehcnol Bioprocess Eng.

[7] Do KH, Park HM, Kim SK, Yun HS (2018). Production of cis-vaccenic acid-oriented unsaturated fatty acid in *Escherichia coli*. Biotechnol Bioprocess Eng.

[8] Choi SY, Rhie MN, Kim HT, Joo JC, Cho IJ, Son J, Jo SY, Sohn YJ, Baritugo KA, Pyo J, Lee YJ, Lee SY, Park SJ (2020). Metabolic engineering for the synthesis of polyesters: a 100-year journey from polyhydroxyalkanoates to non-natural microbial polyesters. Metab Eng.

[9] Sohn YJ, Kim HT, Baritugo KA, Song HM, Ryu MH, Kang KH, Jo SY, Kim H, Kim YJ, Choi J, Park SK, Joo JC, Park SJ (2020). Biosynthesis of polyhydroxyalkanoates from sucrose by metabolically engineered *Escherichia coli* strains. Int J Biol Macromol.

[10] Shelp BJ, Bown AW, McLean MD (1999). Metabolism and functions of gamma-aminobutyric acid. Trends Plant Sci.

[11] Boonstra E, de Kleijn R, Colzato LS, Alkemade A, Forstmann BU, Nieuwenhuis S (2015). Neurotransmitters as food supplements: the effects of GABA on brain and behavior. Front Psychol.

[12] Chua J-Y, Koh MKP, Liu S-Q (2019). Gamma-aminobutyric acid: a bioactive compound in foods. Sprouted grains.

[13] Kim S-H, Shin B-H, Kim Y-H, Nam S-W, Jeon S-J (2007). Cloning and expression of a full-length glutamate decarboxylase gene from *Lactobacillus brevis* BH2. Biotechnol Bioprocess Eng.

[14] Ting Wong CG, Bottiglieri T, Snead OC (2003). GABA, γ-hydroxybutyric acid, and neurological disease. Ann Neurol.

[15] Park SJ, Kim EY, Noh W, Oh YH, Kim HY, Song BK, Cho KM, Hong SH, Lee SH, Jegal J (2013). Synthesis of nylon 4 from gamma-aminobutyrate (GABA) produced by recombinant *Escherichia coli*. Bioprocess Biosyst Eng.

[16] Saskiawan I (2008). Biosynthesis of polyamide 4, a biobased and biodegradable polymer. Microbiol Indones.

[17] Le Vo TD, Kim TW, Hong SH (2012). Effects of glutamate decarboxylase and gamma-aminobutyric acid (GABA) transporter on the bioconversion of GABA in engineered *Escherichia coli*. Bioprocess Biosyst Eng.

[18] Le Vo TD, Ko J-s, Lee SH, Park SJ, Hong SH (2013). Overexpression of *Neurospora crassa* OR74A glutamate decarboxylase in *Escherichia coli* for efficient GABA production. Biotechnol Bioprocess Eng.

[19] Le Vo TD, Ko J-s, Lee SH, Park SJ, Hong SH (2014). Improvement of gamma-amino butyric acid production by an overexpression of glutamate decarboxylase from *Pyrococcus horikoshii* in *Escherichia coli*. Biotechnol Bioprocess Eng.

[20] Plokhov AY, Gusyatiner M, Yampolskaya T, Kaluzhsky V, Sukhareva B, Schulga A (2000). Preparation of γ-aminobutyric acid using *E. coli* cells with high activity of glutamate decarboxylase. Appl Biochem Biotechnol.

[21] Yu P, Chen K, Huang X, Wang X, Ren Q (2018). Production of γ-aminobutyric acid in *Escherichia coli* by engineering MSG pathway. Prep Biochem Biotechnol.

[22] Yu P, Ren Q, Wang X, Huang X (2019). Enhanced biosynthesis of γ-aminobutyric acid (GABA) in *Escherichia coli* by pathway engineering. Biochem Eng J.

[23] Zhao A, Hu X, Li Y, Chen C, Wang X (2016). Extracellular expression of glutamate decarboxylase B in *Escherichia coli* to improve gamma-aminobutyric acid production. AMB Expr.

[24] Zhao W-r, Huang J, Peng C-l, Hu S, Ke P-y, Mei L-h, Yao S-j (2014). Permeabilizing *Escherichia coli* for whole cell biocatalyst with enhanced biotransformation ability from l-glutamate to GABA. J Mol Catal B Enzym.

[25] Pham VD, Lee SH, Park SJ, Hong SH (2015). Production of gamma-aminobutyric acid from glucose by introduction of synthetic scaffolds between isocitrate dehydrogenase, glutamate synthase and glutamate decarboxylase in recombinant *Escherichia coli*. J Biotechnol.

[26] Okai N, Takahashi C, Hatada K, Ogino C, Kondo A (2014). Disruption of *pknG* enhances production of gamma-aminobutyric acid by *Corynebacterium glutamicum* expressing glutamate decarboxylase. AMB Express.

[27] Shi F, Jiang J, Li Y, Li Y, Xie Y (2013). Enhancement of γ-aminobutyric acid production in recombinant *Corynebacterium glutamicum* by co-expressing two glutamate decarboxylase genes from *Lactobacillus brevis*. J Ind Microbiol Biotechnol.

[28] Shi F, Li Y (2011). Synthesis of γ-aminobutyric acid by expressing *Lactobacillus brevis*-derived glutamate decarboxylase in the *Corynebacterium glutamicum* strain ATCC 13032. Biotechnol Lett.

[29] Somasundaram S, Lee SH, Park SJ, Hong SH (2016). Efficient production of gamma-aminobutyric acid using *Escherichia coli* by co-localization of glutamate synthase, glutamate decarboxylase, and GABA transporter. J Ind Microbiol Biotechnol.

[30] Somasundaram S, Lee SH, Park SJ, Hong SH (2016). Gamma-aminobutyric acid production through GABA shunt by synthetic scaffolds introduction in recombinant *Escherichia coli*. Biotechnol Bioprocess Eng.

[31] Zhao A, Hu X, Wang X (2017). Metabolic engineering of *Escherichia coli* to produce gamma-aminobutyric acid using xylose. Appl Microbiol Biotechnol.

[32] Choi JW, Yim SS, Lee SH, Kang TJ, Park SJ, Jeong KJ (2015). Enhanced production of gamma-aminobutyrate (GABA) in recombinant *Corynebacterium glutamicum* by expressing glutamate decarboxylase active in expanded pH range. Microb Cell Fact.

[33] Krisko A, Radman M (2013). Biology of extreme radiation resistance: the way of *Deinococcus radiodurans*. Cold Spring Harb Perspect Biol.

[34] Liu Y, Zhou J, Omelchenko MV, Beliaev AS, Venkateswaran A, Stair J, Wu L, Thompson DK, Xu D, Rogozin IB (2003). Transcriptome dynamics of *Deinococcus radiodurans* recovering from ionizing radiation. Proc Natl Acad Sci USA.

[35] Appukuttan D, Singh H, Park S-H, Jung J-H, Jeong S, Seo HS, Choi YJ, Lim S (2016). Engineering synthetic multistress tolerance in *Escherichia coli* by using a deinococcal response regulator, DR1558. Appl Environ Microbiol.

[36] Guo S, Yi X, Zhang W, Wu M, Xin F, Dong W, Zhang M, Ma J, Wu H, Jiang M (2017). Inducing hyperosmotic stress resistance in succinate-producing *Escherichia coli* by using the response regulator DR1558 from *Deinococcus radiodurans*. Process Biochem.

[37] Park S, Sohn YJ, Park SJ, Choi J (2020). Enhanced Production of 2,3-Butanediol in Recombinant *Escherichia coli* Using Response Regulator DR1558 Derived from *Deinococcus radioduran*. Biotehcnol Bioprocess Eng.

[38] Park S-h, Kim GB, Kim HU, Park SJ, Choi J-i (2019). Enhanced production of poly-3-hydroxybutyrate (PHB) by expression of response regulator DR1558 in recombinant Escherichia coli. Int J Biol Macromol.

[39] Lawson A, Quinn A (1967). Studies on amino acid decarboxylases in *Escherichia coli*. Biochem J.

[40] Shukuya R, Schwert GW (1960). Glutamic acid decarboxylase I Isolation procedures and properties of the enzyme. J Biol Chem.

[41] Somasundaram S, Maruthamuthu MK, Ganesh I, Eom GT, Hong SH (2017). Enchancement of gamma-aminobutyric acid production by co-localization of *Neurospora crassa* OR74A glutamate decarboxylase with *Escherichia coli* GABA transporter via synthetic scaffold complex. J Microbiol Biotechnol.

[42] Foster JW (2004). *Escherichia coli acid* resistance: tales of an amateur acidophile. Nat Rev Microbiol.

[43] Kanjee U, Houry WA (2013). Mechanisms of acid resistance in *Escherichia coli*. Annu Rev Microbiol.

[44] Castanie-Cornet M-P, Penfound TA, Smith D, Elliott JF, Foster JW (1999). Control of acid resistance in *Escherichia coli*. J Bacteriol.

[45] Iyer R, Williams C, Miller C (2003). Arginine-agmatine antiporter in extreme acid resistance in *Escherichia coli*. J Bacteriol.

[46] Kanjee U, Gutsche I, Alexopoulos E, Zhao B, El Bakkouri M, Thibault G, Liu K, Ramachandran S, Snider J, Pai EF (2011). Linkage between the bacterial acid stress and stringent responses: the structure of the inducible lysine decarboxylase. EMBO J.

[47] Kashiwagi K, Suzuki T, Suzuki F, Furuchi T, Kobayashi H, Igarashi K (1991). Coexistence of the genes for putrescine transport protein and ornithine decarboxylase at 16 min on *Escherichia coli* chromosome. J Biol Chem.

[48] Feehily C, Karatzas K (2013). Role of glutamate metabolism in bacterial responses towards acid and other stresses. J Appl Microbiol.

[49] Chattopadhyay MK, Keembiyehetty CN, Chen W, Tabor H (2015). Polyamines stimulate the level of the σ38 subunit (RpoS) of *Escherichia coli* RNA polymerase, resulting in the induction of the glutamate decarboxylase-dependent acid response system via the *gadE* regulon. J Biol Chem.

[50] Seo SW, Kim D, O’Brien EJ, Szubin R, Palsson BO (2015). Decoding genome-wide GadEWX-transcriptional regulatory networks reveals multifaceted cellular responses to acid stress in *Escherichia coli*. Nat Commun.

[51] Krulwich TA, Sachs G, Padan E (2011). Molecular aspects of bacterial pH sensing and homeostasis. Nat Rev Microbiol.

[52] Nishio Y, Ogishima S, Ichikawa M, Yamada Y, Usuda Y, Masuda T, Tanaka H (2013). Analysis of l-glutamic acid fermentation by using a dynamic metabolic simulation model of *Escherichia coli*. BMC Syst Biol.

[53] Ginésy M, Rusanova-Naydenova D, Rova U (2017). Tuning of the carbon-to-nitrogen ratio for the production of l-arginine by *Escherichia coli*. Fermentation (Basel).

[54] Kim JM, Lee KH, Lee SY (2017). Development of a markerless gene knock-out system for *Mannheimia succiniciproducens* using a temperature-sensitive plasmid. FEMS Microbiol Lett.

[55] Palmeros Bz, Wild J, Szybalski W, Le Borgne S, Hernández-Chávez G, Gosset G, Valle F, Bolivar F (2000). A family of removable cassettes designed to obtain antibiotic-resistance-free genomic modifications of *Escherichia coli* and other bacteria. Gene.

[56] Soma Y, Fujiwara Y, Nakagawa T, Tsuruno K, Hanai T (2017). Reconstruction of a metabolic regulatory network in *Escherichia coli* for purposeful switching from cell growth mode to production mode in direct GABA fermentation from glucose. Metab Eng..

[57] Datsenko KA, Wanner BL (2000). One-step inactivation of chromosomal genes in *Escherichia coli* K-12 using PCR products. Proc Natl Acad Sci USA.

[58] Kim YH, Kim HJ, Shin J-H, Bhatia SK, Seo H-M, Kim Y-G, Lee YK, Yang Y-H, Park K (2015). Application of diethyl ethoxymethylenemalonate (DEEMM) derivatization for monitoring of lysine decarboxylase activity. J Mol Catal B Enzym..

[59] Livak KJ, Schmittgen TD (2001). Analysis of relative gene expression data using real-time quantitative PCR and the 2^− ΔΔCT^ method. Methods..

