# Supplementary material for: Effect of DR1558, a Deinococcus radiodurans response regulator, on the production of GABA in the recombinant Escherichia coli under low pH conditions
Source: Microb Cell Fact. 2020 Mar 10;19:64. doi: 10.1186/s12934-020-01322-3 (PMC7063819; doi:10.1186/s12934-020-01322-3)
Supplement: Supplementary file 1 — Additional file 1: Table S1. Primers used in this study. Table S2. Relative gene expression of dr1558 overexpression strain. Figure S1. Time profile of GABA and pH in flask cultivation of recombinant E. coli. A) E. coli DGB201 strain expressing GdhA and GadBC genes.; B) E. coli DGB202 strain expressing IcdA, GdhA, and GadBC genes. Strains were cultivated in 50 mL MR medium supplied with 5 g/L Yeast Extract, 30 g/L glucose in a baffled flask at 30 °C for 24 h. [file 12934_2020_1322_MOESM1_ESM.docx]

**Additional Materials**

**Effect of DR1558, a *Deinococcus radiodurans* response regulator, on the production of GABA in the recombinant *Escherichia coli* under low pH conditions**

Sung-ho Park^1^, Yu Jung Sohn^2^, Si Jae Park^2,^*, Jong-il Choi^1,^*

^1^Department of Biotechnology and Bioengineering, Interdisciplinary Program for Bioenergy & Biomaterials, Chonnam National University, 77 Yongbong-ro, Gwangju 61186, Republic of Korea

^2^Division of Chemical Engineering and Materials Science, Ewha Womans University, 52 Ewhayeodae-gil, Seodaemun-gu, Seoul 03760, Republic of Korea

*Corresponding authors

E-mail addresses: choiji01@jnu.ac.kr (J. Choi), parksj93@ewha.ac.kr (S.J. Park)

**Table S1.** Primers used in this study.

| **Primers for quantitative real-time PCR** | | | | | | |
| --- | --- | --- | --- | --- | --- | --- |
| Pathway | Gene | | | Forward primer | Reverse primer | |
| Internal control | polA | | GCTGAACGTGCAGCCATTAA | | CAATCATCGCCCGTTTGATAA | |
|  | dnaA | | TGGCGAAAGAGCTGACTAACC | | ACGGCAGGCATGAAGCA | |
| Sigma factors | rpoD | | TGCGTATGCGTTTCGGTATC | | GCGGGTAACGTCGAACTGTT | |
|  | rpoS | | CGCCGCCGGATGATC | | CAGACCACGATTGCCATAACG | |
| Transcription factors | ihfA | | CGAAAACGGGCGAGGATA | | CGACCCGGCTTTTTAACTTCT | |
|  | Crp | | TGTTTGAAGAGGGCCAGGAA | | CCACTTCACAGGCGGTTTTC | |
|  | pdhR | | CATCGCCATCTGGCCTTTAT | | CACGGCGGCTCTCTTCA | |
|  | fruR | | GGTTGGTGCCGATCAGGAT | | TCTCGGCGGGAAACTTACG | |
| PPP | zwf | | GGCGCTGCGTTTTGCTAA | | TTCTGCCACGGTAATCTCAACA | |
| Glucose transport | ptsG | | ACTGGCGTTGTGCTGTCCTT | | GGTAAGCAGCCCACTGAGAGA | |
| Glycolysis | pykF | | CGAGTTCAACAATGACAACCGTAA | | ATCAGCGGAGCATCCAGTTT | |
|  | pykA | | GCGCTGACCGAAAAAGACAA | | CAGCCAGGTAATCTACGCCAAT | |
|  | aceE | | CCAAAGGCAAAGCGACAGTAAT | | CCTGGTGCGCGATGTTTT | |
| Acetate metabolism | acs | | AACACACCATTCCTGCCAACA | | TGTTGATACATCGCCTCGTACTG | |
|  | actP | | ACCGGGTTTATGGGCTACTTCTA | | CGGATTCGCACCAACCA | |
|  | ackA | | CGCGCAATGGACGTTTACT | | TCCAGACGACCATCCATCAG | |
|  | poxB | | AGCGTGCTGGGCTTTGTG | | TAGTTCGGTGCCGTCAGTCA | |
|  | pta | | GTCCTGACCTGATGATCGAC | | AACGCTGTACCGCTTTGTAG | |
| TCA cycle | icdA | | AAGTTCACCGAAGGAGCGTTTA | | GCCACCGTCGATCAGTTCA | |
|  | sucA | | GTCTCGCGTTGCCAAGATTTAT | | GTGGCGTAAGCGAGGTTTTC | |
|  | sdhC | | CACATGATGATGGATTTTGGCTAT | | GCACGACAGTAATAACAAAGGAGATTT | |
|  | mdh | | AGGCGCTTGCACTACTGTTAAAA | | CGGGAGTCACTGGAGCGATA | |
| Glyoxylate shunt | aceA | | TGCACGGTGAGTCGAAAAAA | | TAGACTGCTTCAATACCCGCTTT | |
|  | aceB | | TGGCGTGGTGAGGCAAT | | GGAAGAAATAGAGCGCAAAATCA | |
| Respiratory chain complexes | nuoM | | GCTGAAAACTGCCGCTTAC | | GAAGATGCCGATAACACCC | |
|  | nuoN | | TTATCGCCTTTGCCTCCATC | | AAACCCCTACCGCTTCCATC | |
|  | cyoA | | TCAAAGTGACCTCCAACTCC | | ACTTCATGCCTGAGAAGCC | |
|  | cyoB | | CAAACGATGAACGACAGCAC | | TCTCCGAAATTGCGGCAAC | |
|  | cyoC | | AACGCCAGCCAGGAGATAAC | | TGTTTGCTACCTATGCCGTTC | |
|  | cyoD | | ACACCAGATGCACCAGAACC | | GCAGCGTAAAAACCTACATGAC | |
| Glutamate decarboxylase acid resistance (GDAR) | gadA | | ATATCCGCAATCCGCAGCCATC | | CCAACGCCATTTCATCGCCATC | |
|  | gadB | | ATATCCGCAATCCGCAGCCATC | | CCAACGCCATTTCATCGCCATC | |
|  | gadC | | TCTCCGCAGGGGTAATGCAAAC | | ACATACATCCCGCGAGAAGGAC | |
|  | gadE | | TGGCACCTTATCACATCAGT | | ATGGGGCAAGTGTTTACCAT | |
|  | gadX | | TTATGGGATGACGCCCACAGAG | | ATTCCTTCCGCAGAACGGTCAG | |
|  | gadW | | TGCGTTTTGTTCACCGGATACG | | TCAAAAATCGCCGTCACCAGC | |
|  | ybaS | | TTACAGCAGGCAGTGGATCAGG | | TTGCCATCGCAGGTCACGATAG | |
| **Primers for gene cloning** | | | | | | |
| Gene | | Forward primer | | | | Reverse primer |
| dr1558 | | | ATTATATACCATGGATGACTCTGCCTCAAGGAGAA | | TATATAATGGATCCTCACAACTCCACGCCCTCCAG | |
| icdA | | | ATTAGCATATGATGGAAAGTAAAGTAGTTGTTCCG | | ATTACAGATCTTTACATGTTTTCGATGATCGCGT | |
| gdhA | | | ATGGATCAGACATATTCTCTGGAGTC | | ATTACGCTCGAGTTAAATCACACCCTGCGCCA | |
| **Primers for gene knock out** | | | | | | |
| Gene | | | Forward primer | | Reverse primer | |
| gabT | | ATGAGCAGCAATAAAGAGTTAATGCAGCGCCG  CAGTCAGGCGATTCCCCGTACCGTTCGTATAAT  GTATG | | | | CTACTGCTTCGCCTCAGCAAAACACTGGCTGATGATCTCCAGACCCTGACTACCGTTCGTATAGCATAC |
| sucA | | ATGCAGAACAGCGCTTTGAAAGCCTGGTTGGAC  TCTTCTTACCTCTCTGGTACCGTTCGTATAATGT  ATG | | | | TTATTCGACGTTCAGCGCGTCATTAACCAGATCTTGTTGCTGTTTCTGGTTACCGTTCGTATAGCATAC |

**Table S2.** Relative gene expression of *dr1558* overexpression strain.

|  | gene | pathway | Relative expression level | *P* value |
| --- | --- | --- | --- | --- |
| *rpoD* | RNA polymerase sigma 70 subunit | Transcriptional regulators | 2.35$\pm$0.18 | 3.63E-05 |
| *rpoS* | RNA polymerase sigma 38 subunit |  | 1.87$\pm$0.19 | 1.04E-04 |
| *ihfA* | Integration host factor |  | 1.1$\pm$0.15 | 4.80E-04 |
| **Crp* | cAMP repression protein |  | 0.18$\pm$0.15 | 1.45E-01 |
| *pdhR* | Pyruvate dehydrogenase complex regulator |  | 1.99$\pm$0.16 | 1.40E-04 |
| **fruR* | cAMP independent protein |  | 0.16$\pm$0.25 | 5.12E-01 |
| *zwf* | Glucose 6-phosphate dehydrogenase | Glycolysis | 0.32$\pm$0.09 | 1.33E-02 |
| *ptsG* | Subunit of Enzyme IIglc |  | 2.1$\pm$0.09 | 1.88E-05 |
| *pykF* | Pyruvate kinase |  | 0.78$\pm$0.1 | 3.05E-03 |
| *pykA* | Pyruvate kinase |  | 1.76$\pm$0.08 | 4.36E-04 |
| *aceE* | Pyruvate dehydrogenase E1 componenet |  | 0.61$\pm$0.15 | 2.36E-03 |
| *acs* | Acetyl-CoA synthetase | Acetate metabolism | -0.78$\pm$0.06 | 9.72E-03 |
| **actP* | Acetate Permease |  | -0.17$\pm$0.1 | 2.7E-01 |
| **ackA* | Acetate kinase |  | 0.09$\pm$0.08 | 2.2E-01 |
| *pta* | phosphotransacetylase |  | 0.13$\pm$0.1 | 7.66E-03 |
| *poxB* | pyruvate oxidase |  | -3.67$\pm$0.08 | 9.30E-05 |
| *icdA* | Isocitrate dehydrogenase | TCA Cycle | 1.11$\pm$0.27 | 7.68E-03 |
| *sucA* | 2-oxoglutarate dehydrogenase subunit |  | 2.47$\pm$0.25 | 5.92E-03 |
| *sdhC* | succinate dehydrogenase |  | 1.16$\pm$0.25 | 4.50E-02 |
| **mdh* | malate dehydrogenase |  | 0.19$\pm$0.26 | 7.56E-01 |
| *aceA* | Isocitrate lyase | Glyoxylate shunt | 2.36$\pm$0.28 | 4.11E-03 |
| *aceB* | Malate synthase |  | 2.23$\pm$0.29 | 1.15E-02 |

Logarithmic ratios were determined by the 2^-△△Ct^ method. The *E. coli* BL21(DE3) harboring pH3BN strain was used as control under exponential growth phase. Only those genes whose expression changed significantly, more than 2-fold (*P* <0.05), were considered significant. For all genes represented in this table, the *P*-value was <0.05 except for genes marked with asterisks.


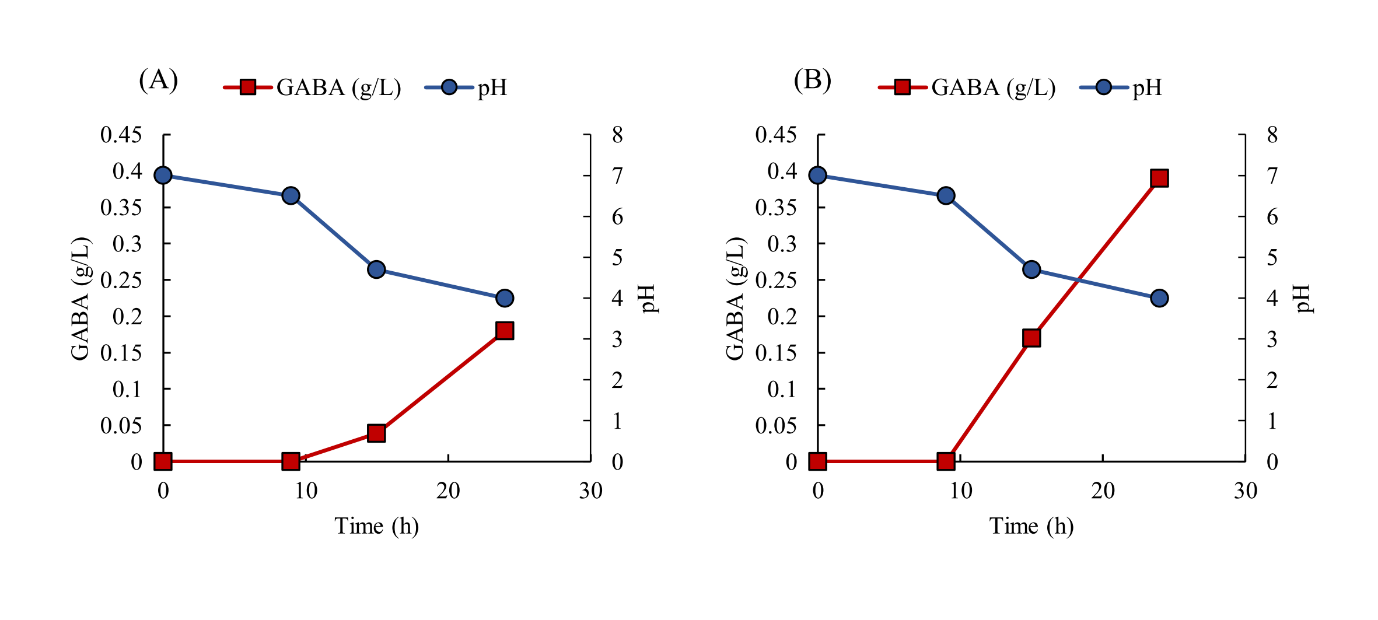


**Figure S1.** Time profile of GABA and pH in flask cultivation of recombinant *E. coli.* A) *E. coli* DGB201 strain expressing GdhA and GadBC genes.; B) *E. coli* DGB202 strain expressing IcdA, GdhA, and GadBC genes. Strains were cultivated in 50 mL MR medium supplied with 5 g/L Yeast Extract, 30 g/L glucose in a baffled flask at 30 ^o^C for 24 h.
